# Advances in the Astonishing World of Phytochemicals: State-of-the-Art for Antioxidants—2nd Edition

**DOI:** 10.3390/antiox14050582

**Published:** 2025-05-12

**Authors:** Marianna Lauricella, Antonella D’Anneo

**Affiliations:** 1 Section of Biochemistry, Department of Biomedicine, Neurosciences and Advanced Diagnostics (BIND), University of Palermo, 90127 Palermo, Italy; 2 Laboratory of Biochemistry, Department of Biological, Chemical and Pharmaceutical Sciences and Technologies (STEBICEF), University of Palermo, 90127 Palermo, Italy

This Editorial refers to the Special Issue titled “Advances in the Astonishing World of Phytochemicals: State-of-the-Art for Antioxidants—2nd Edition”, which highlights the multifaceted properties of natural compounds containing antioxidants and describes the need to understand how active compounds, solvents, and complex formations interact, in order to better establish their potential in applied sciences.

The plant world is a fascinating source of biodiversity and physiologically active substances with antioxidant properties, which help to neutralize free radicals and reduce oxidative stress. Polyphenols are one of the main phytoconstituents, with antioxidant potential found in different parts of plants, including flowers, leaves, fruits, roots, and seeds [1]. Moreover, polyphenols are a diverse group of naturally occurring compounds that play a significant role in protecting plants from environmental stressors and pathogens [2].

Antioxidants derived from plant sources have a wide range of applications across various fields, including health, nutrition, cosmetics, agriculture, and food preservation. These plant-based antioxidants offer natural, sustainable, and often safer alternatives to synthetic chemicals, making them valuable in the development of new products [3,4].

In cosmetics, antioxidants derived from plants are widely used in the preparation of skincare products [5]. Exposure to UV rays and free radicals significantly contributes to skin damage, with the formation of wrinkles and dark spots hastening aging by interfering with defense and restorative processes. The plant world is a rich and diverse source of compounds and nutricosmetics that can play a key role in promoting health and skin vitality, and these have received a great deal of attention from pharmaceutical companies for their anti-free radical potential.

Some common plant-based antioxidants include flavonoids, polyphenols, and vitamins such as Vitamin C and E. In the beauty industry, plant-based ingredients can be used as supplements, nutricosmetics, and cosmetics in the preparation of skincare products with radical scavenging activity in order to create a skin-protective shield from UV rays and free radicals (contribution 1).

Plant-derived compounds also have remarkable uses in agriculture, benefiting both crop production and plant protection due to their antioxidant and antimicrobial properties. Particular interest has been paid to the application of antioxidants in horticulture as plant biostimulants are able to ameliorate stress-associated injury, favor plant growth, increase fruit quality, and reduce toxicity risks. On this topic, research conducted by Vichi et al. (contribution 2) proved that a zinc–caffeine-combined application was able to promote metabolic processes, enriching the nutritional value of high-quality fruits, as well as improving plant growth. Indeed, the authors evaluated the effect of zinc, caffeine, and a combination of these administered in the irrigation procedures of *Solanum lycopersicum* L. plants. The research provided evidence that the zinc-caffeine combination could activate the phenylpropanoid pathway of tomatoes, not only increasing the content of 4-coumaric acid, caffeic acid, and t-ferulic acid but also enhancing the antioxidant properties of the fruits. The analysis of mineral element content assayed in the tomatoes obtained from *Solanum lycopersicum* L. plants that were cultivated with this procedure also demonstrated the absence of health risks after consumption of these fruits. The study is in line with the strategies set by the Food and Agriculture Organization (FAO) of the United Nations that support sustainable agricultural approaches aiming to increase food production and also reduce environmental impacts.

As reported by del Carmen Villegas-Aguilar in her contribution, plant-derived extracts rich in phenolic compounds possess a different plethora of bioactive properties (contribution 3). Indeed, for some classes of phenolic compounds, exact structure–bioactivity relationship mechanisms still remain unknown. However, analyzing five plant matrices (*Theobroma cacao*, *Hibiscus sabdariffa*, *Silybum marianum*, *Lippia citriodora*, *and Olea europaea*) with different phenolic compositions the authors provided evidence that *T. cacao* and *S. marianum* exerted similar inhibitory effects in the enzymes involved in phlogosis processes, as well as in skin aging, highlighting the fact that shared bioactive properties among phenolic compounds can be identified in different matrices.

Different plant extracts have been studied for their potential to treat cancer, and many of them have shown promising effects. *Ganoderma lucidum*, a mushroom known under different names depending on the country, has been extensively used in traditional medicine, particularly in East Asia, for its health benefits. On this topic, Cadar’s review highlights the bio-compounds in *Ganoderma lucidum* that have been shown to have cancer-fighting effects (contribution 4). These bioactive molecules include polysaccharides, triterpenoids, sterols, proteins, nucleotides, fatty acids, vitamins, and minerals. These compounds have demonstrated multiple anticancer effects, namely immunomodulatory, anti-proliferative, cytotoxic, and antioxidant actions. However, there is uncertainty about the effects of *Ganoderma lucidum* bio-compounds in the treatment of different cancers. Such an effect could be ascribed to variations in the types of *Ganoderma lucidum* used, differences in the people studied, or interactions between the mushroom and other treatments. Thus, more rigorous and standardized clinical research is necessary to confirm its therapeutic potential and to better understand the mechanisms underlying its anticancer effects.

Apart from studies on plant-derived extracts, research on the nutritional value and antioxidant potential of fruits has gained particular attention due to the important role that fruits play in human health. On this topic, the nutritional value and antioxidant potential of *Solanum lycopersicum* L. fruits were investigated by Woo Baek et al. (contribution 5), who, in their analysis of the properties of five cherry tomato cultivars (green-colored Jocheong, yellow-colored BN Satnolang, orange-colored Gold Chance, black-colored Black Q, and red-colored Snacktom cherry tomatoes), measured physicochemical parameters and compared the firmness, color values, titratable acidity, brix-to-acid ratio (BAR), and total soluble solids of the fruits. Their analysis showed relevant changes in the BAR, as well as in some metabolite profiles, of the content of some amino acids, thus providing evidence for the different taste among cherry tomato cultivars, with metabolite content related to fruit color. In addition, the cherry tomato cultivars analyzed were shown to possess different amounts of pigment content, such as anthocyanins, chlorophylls, lycopene, and β-carotene contents, which could favorably impact consumers’ taste. On the other hand, the presence of lycopene and β-carotene, as well as chlorophylls, sustained the remarkable antioxidant properties of the fruit. The research proposed by these authors emphasizes the relevance of a cultivar screening program to ameliorate the nutritional profile and promote the inclusion of different tomato varieties in order to obtain additive or synergistic effects in secondary metabolite combinations.

Another functional food is *Citrus*, which possesses healthy properties due to the presence of carotenoids, a large family of isoprenoid pigments with antioxidant functions [6]. The chemical profiles of carotenoids change in different cultivars. In rare cases, *Citrus* fruits accumulate lycopene, a potent antioxidant carotene, which confers major nutritional and nutraceutical value to the fruits. Lycopene is present in red-orange fruits, whereas it is absent in orange fruits. Characterizing new red-orange cultivars could constitute a strategy for the citrus industry to diversify the color range and enhance the nutritional value of orange juice. In their study, Carmona et al. (contribution 6) characterized the fruit quality and biochemical features of two new red-fleshed sweet orange varieties, Carrancas and Pinhal, both originating in Sao Paulo (Brazil). In both of these cultivars, the authors demonstrated a high accumulation of lycopene and β-carotene carotenoids, thus highlighting their possible addition to the juice of popular blond-orange fruits.

*Theobroma cacao* L., commonly known as cocoa, has played an important role in human culture and diet for thousands of years. Sitarek’s paper highlights how it also possesses therapeutic potential, relying on its anti-inflammatory and anticancer properties, which come from its polyphenol, flavonoid, and alkaloid content (contribution 7). *Theobroma cacao* L. extracts have been shown to have significant effects in cancer treatment, such as slowing tumor growth, inducing cancer cell death, and possibly preventing metastasis in various cancer cell lines and animal models. Moreover, cacao extracts demonstrated potential synergistic effects when combined with chemotherapy drugs to improve their effectiveness and reduce the organ toxicity caused by chemotherapy. Research into combining cacao extracts with traditional treatments could lead to new possibilities in cancer therapy and the treatment of chronic inflammatory diseases. Furthermore, combining cacao extracts with nanotechnology could be a promising direction for research in the future, as nanoparticles can improve the bioavailability, target delivery, and controlled release of active compounds. Future studies should focus on optimizing nanoparticle-based systems, examining their safety, and testing their long-term effectiveness for different treatments.

The importance and possible impact of antioxidants have been highlighted, specifically for when an imbalance between the body’s natural defense systems and the generation of reactive oxygen species occurs, which promotes the development of chronic illnesses associated with oxidative stress, such as cancer, cardiovascular diseases, and neurodegenerative diseases (contribution 8). For these reasons, the potential of antioxidants has attracted the interest of researchers in nanotechnology, thus offering cutting-edge strategies to enhance drug delivery and promising revolutionary advancements in healthcare (contribution 8). In such a direction, green nanotechnology not only allows for the development of nanoparticle-based strategies to protect flavonoids from degradation in the gastrointestinal tract, but it also makes the creation of controlled release systems possible, maintaining these molecules in the bloodstream for prolonged periods in order to exert their proper therapeutic potential.

Natural antioxidants may play a significant role in managing rheumatoid arthritis (RA), a systemic autoimmune disease primarily affecting the synovial joints and defined by concomitant systemic inflammation and autoantibody production [7]. Although the availability of biological immunosuppressive and immunomodulatory agents alone or in association with nonsteroidal anti-inflammatory drugs or glucocorticoids has significantly improved the course of the disease in patients with RA, patients often do not adequately respond to current treatment regimens due to tolerance development or severe side effects. Thus, there is an urgent need for therapeutic management using “safe” molecules. Olive oil, especially extra virgin olive oil (EVOO), is renowned for its numerous health benefits, largely attributed to its rich composition of monounsaturated fats and bioactive compounds such as phenolic compounds [8]. A study by Tamburini et al. (contribution 9) highlights the anti-inflammatory and antioxidant effects of extra virgin olive oil polyphenol-enriched extracts (PE-EVOOs) on an RA model. In their study, the authors demonstrated how the treatment of peripheral mononuclear cells (PBMCs) obtained from RA patients with PE-EVOOs resulted in a reduction in intracellular reactive oxygen species (ROS) and pro-inflammatory cytokines, such as tumor necrosis factor-α (TNF-α) and interleukin-1 β (IL-1β). These effects seem to be related to the inhibition of the phosphorylated and active forms of the inflammatory transcription factor NF-κB, as well as to an upregulation of the transcription factor nuclear factor erythroid-2-related factor 2 (Nrf2) and its target antioxidant enzyme catalase and manganese superoxide dismutase (MnSOD). Collectively, these results suggest a possible use of PE-EVOOs as potential adjuvants for the treatment of RA.

Antioxidant potential has also been recognized in olive leaves, the main byproducts generated from olive tree cultivation and processing, due to the presence of phenolic compounds [9]. These are both present in free and bound forms. In their paper, Li et al. (contribution 10) characterizes both free phenolics (FBs) and bound phenolics (BPs) in olive leaves and compares their antioxidant activity in both in vitro and in vivo models. While FBs exhibited significantly higher antioxidant activity than BPs in chemical antioxidant assays (DPPH, ABTS, and FRAP assays), similar antioxidant effects were observed for FBs and BPs in biological systems (cellular and in vivo mouse models). The authors demonstrated that both FPs and BPs protected HepG2 cells from H_2_O_2_-induced oxidative injury and were effective in mitigating oxidative damage by restoring SOD, CAT, and GSH-Px activities in aging mice. FPs exert their antioxidant activity partly via the activation of the Nrf2 signaling pathway; regarding BP mechanisms, the overall scenario seems to be unclear, and further studies are needed.

Indeed, beyond fruits, the plant kingdom offers a vast array of plants with healthy properties. However, the limited solubility of most of their compounds limits their potential clinical application [10]. To improve the bioavailability of nutraceuticals, the pharmaceutical industry has attempted to encapsulate them in β-cyclodextrin complexes [11]. *Inula sarana* is a plant of the Asteraceae family, which is largely spread in Asia, Europe, and Africa [12]. It exhibits antioxidant, anti-inflammatory, and anticancer properties due to the presence of different phytocompounds such as polyphenols, diterpenoids, and flavonoids [13]. Zengin et al.’s study aimed to compare the different compositions and properties of *Inula sarana* extracts in different solvents, including n-hesane, ethyl acetate, dichloromethane, 70% ethanol, and water (contribution 11). The properties of the different extracts were also compared with those incorporated into β-cyclodextrin. The authors demonstrated that water and 70% ethanol extracts had the highest phenolic content and highest antioxidant properties among the extracts analyzed. Additionally, ethanol and hexane extracts displayed the highest inhibition levels out of the different enzymes analyzed, while ethyl acetate extracts contained high levels of flavonoids. Notably, the inclusion complex displayed relatively little or no antioxidant efficacy and enzyme inhibitory potential compared to pure extracts.

Following the rigorous *Antioxidants* review process, eleven papers (seven manuscripts and four reviews in all) were accepted for the publication in this Special Issue. All of these contributions are listed below.
